# Anti-Inflammatory Activity of Peptides from *Ruditapes philippinarum* in Lipopolysaccharide-Induced RAW264.7 Cells and Mice

**DOI:** 10.3390/foods13060883

**Published:** 2024-03-14

**Authors:** Haisheng Lin, Weiqiang Shen, Yu Jiang, Qihang Wu, Jialong Gao, Wenhong Cao, Huina Zheng, Zhongqin Chen, Saiyi Zhong, Xiaoming Qin

**Affiliations:** 1 Guangdong Provincial Key Laboratory of Aquatic Products Processing and Safety, Guangdong Provincial Engineering Technology Research Center of Seafood, Guangdong Province Engineering Laboratory for Marine Biological Products, National Research and Development Branch Center for Shellfish Processing (Zhanjiang), College of Food Science and Technology, Guangdong Ocean University, Zhanjiang 524088, China; linhs@gdou.edu.cn (H.L.); shenweiqiang1@stu.gdou.edu.cn (W.S.); jiangyuhello@163.com (Y.J.); 2112203066@stu.gdou.edu.cn (Q.W.); gaojl@gdou.edu.cn (J.G.); cwenhong@gdou.edu.cn (W.C.); zhenghn@gdou.edu.cn (H.Z.); chenzhongqin@gdou.edu.cn (Z.C.); zhongsy@gdou.edu.cn (S.Z.); 2 Shenzhen Institute of Guangdong Ocean University, Shenzhen 518108, China; 3 Collaborative Innovation Center of Seafood Deep Processing, Dalian Polytechnic University, Dalian 116034, China

**Keywords:** *Ruditapes philippinarum* peptides, RAW264.7 cells, in vitro, in vivo, anti-inflammatory, lipopolysaccharide

## Abstract

In our previous study, two peptides with favorable anti-inflammatory effects, Asp-Gln-Thr-Phe (DQTF) and Gly-Tyr-Thr-Arg (GYTR), were screened from *Ruditapes philippinarum* using an in vitro–in silico strategy. The present study aims to investigate the ameliorative effect of *Ruditapes philippinarum* peptides (RPPs) on acute inflammation and clarify the potential mechanism through in vitro and in vivo experiments. The anti-inflammatory effects of DQTF and GYTR were verified with a lipopolysaccharide (LPS)-induced RAW264.7 cell acute inflammation model and the anti-inflammatory effect of the enzymatic hydrolysates of *Ruditapes philippinarum* was explored in vivo using an LPS-induced acute inflammatory injury model in mice. The results show that DQTF and GYTR improved the morphology of LPS-injured cells and decreased the concentrations of tumor necrosis factor (TNF)-α and interleukin (IL)-6 in LPS-induced cells. Moreover, the antioxidant enzyme activity in cells was markedly increased with DQTF and GYTR. The enzymatic hydrolysates of *Ruditapes philippinarum* were obtained with hydrolysis using pepsin–chymotrypsin–trypsin (PeCTHC) and pepsin–trypsin (PeTHC), respectively. PeCTHC and PeTHC significantly reduced pro-inflammatory cytokines and nitric oxide (NO) in the serum. Additionally, the blood indices and levels of superoxide dismutase (SOD), glutathione peroxidase (GSH-PX), and malondialdehyde (MDA) in the livers of mice were markedly improved with RPPs administration. In conclusion, RPPs have preventive and protective effects on acute inflammation, with significant prospects for development in the field of functional foods.

## 1. Introduction

Inflammation is recognized as a critical contributor to the development of many chronic diseases, with eight of the top ten leading causes of death globally stemming directly or indirectly from inflammatory responses [1]. The inflammatory response is defined as a widespread, comprehensive, and helpful immunologic defense of the body to exogenous injury that is systemic and nonspecific in nature [2]. An excessive inflammatory response leads to the overexpression of pro-inflammatory cytokines (such as tumor necrosis factor (TNF)-α, interleukin (IL)-6, and IL-1β), high generation of nitric oxide (NO), and alterations in cellular morphology and blood indices [3,4,5]. Furthermore, inflammation and oxidative stress are two pathophysiologic processes that occur simultaneously. When the body undergoes an inflammatory reaction, it is associated with a decrease in antioxidant enzyme (such as superoxide dismutase (SOD), glutathione peroxidase (GSH-PX), and catalase (CAT)) levels and an increase in the content of malondialdehyde (MDA) [6].

Currently, the agents widely used to treat inflammation are antibiotics, non-steroidal anti-inflammatory drugs (NSAIDs), and immunosuppressants. Nevertheless, these medications are accompanied by serious adverse effects, such as reduced immune cells, damage to the digestive tract, and nephrotoxicity, and thus, limit their wide application for chronic or prophylactic use [7,8,9]. Consequently, in recent years, the acquisition of anti-inflammatory components (e.g., polysaccharides, alkaloids, and bioactive peptides) from naturally occurring diets has received growing focus due to their safety and non-toxicity [10,11,12]. To date, bioactive peptides from typical edible protein hydrolysates have been demonstrated to have an anti-inflammatory effect in cellular and mouse models. For example, soybean hydrolysate [13], lotus seed protein hydrolysate [14], and abalone hydrolysate [15] have been proven to be effective and safe in alleviating inflammatory responses through the inhibition of pro-inflammatory cytokine generation. Moreover, fish protein peptides [16], gelatin peptides [17], and broccoli seed extracts [18] could restore the levels of antioxidant enzymes, thereby inhibiting inflammatory effects. Notably, over the past few decades, interest in utilizing dietary protein hydrolysates has been rising because of their outstanding anti-inflammatory activity, biocompatibility, and non-toxicity.

*Ruditapes philippinarum*, also known as miscellaneous clams or flower clams, is widely distributed in the north and south of the Chinese sea areas, and it is one of the four major economically farmed shellfish in China [19], which is rich in proteins, minerals, and amino acids. It is a kind of high-protein, low-fat, and high-quality aquatic shellfish [20]. In Oriental folk medicine, *Ruditapes philippinarum* is a marine treasure for preventing illnesses in humans (chronic bronchitis, dry cough, phlegm, etc.) [21]. *Ruditapes philippinarum* has been proven to have antioxidant [22,23], antitumor [24], hepatoprotective [25], hypoglycemic [26,27], hypotensive [22], antimicrobial [28], anticarcinogenic [29], immunomodulatory [21], and other effects, but there are few studies on its anti-inflammatory activity.

Previously, we found two peptides, DQTF (composite enzymatic hydrolysis of pepsin, chymotrypsin, and trypsin) and GYTR (composite enzymatic hydrolysis of pepsin and trypsin), from *Ruditapes philippinarum* using virtual enzymatic hydrolysis and molecular docking tools, and confirmed their favorable anti-inflammatory activities with in vitro cellular assays [30]. However, the in vitro mechanism of action of *Ruditapes philippinarum* peptides (RPPs) is unknown, and the in vivo anti-inflammatory effect has not been verified. Hence, this study investigated the ameliorative effects of RPPs and the possible mechanisms of action of RPPs in a lipopolysaccharide (LPS)-induced acute inflammation model from the perspective of pro-inflammatory cytokines and antioxidant enzyme levels. This study provides a theoretical foundation for the high-value utilization of *Ruditapes philippinarum* and the creation of RPPs in the field of functional foods.

## 2. Materials and Methods

### 2.1. Materials

*Ruditapes philippinarum* was purchased from Zhanjiang (Guangdong, China). DQTF and GYTR were ordered from Shanghai Qiangyao Biotechnology Co., Ltd. (Shanghai, China) [27]. RAW264.7 cells were provided by the cell bank of the Institute of Chinese Academy of Sciences (Shanghai, China). LPS was ordered from Sigma-Aldrich Co., Ltd. (St. Louis, MO, USA). Dexamethasone was offered by Guangdong Nanguo Pharmaceutical Co., Ltd. (Guangdong, China). Bicinchoninic acid (BCA) and NO assay kits were supplied by the Beyotime Institute of Biotechnology (Shanghai, China). The SOD, MDA, CAT, and GSH-PX assay kits were obtained from the Nanjing Jiancheng Institute of Biological Engineering (Nanjing, China). RPMI 1640 and penicillin-streptomycin were provided by Shanghai Canspec Scientific Instruments Co., Ltd. (Shanghai, China). Fetal bovine serum was ordered from Shenzhen Amperex Bio-technology Co., Ltd. (Shenzhen, China). The IL-6, IL-1β, and TNF-α ELISA kits were purchased from Jiangsu Enzyme Exemption Industry Co., Ltd. (Jiangsu, China). Chymotrypsin (1.2 × 10^6^ U/g), trypsin (2.5 × 10^5^ U/g), and pepsin (2.5 × 10^5^ U/g) were obtained from Beijing Solarbio Biotechnology Co., Ltd. (Beijing, China). Bis-urea, glutathione, and trichloroacetic acid (TCA) were provided by Shanghai Yuanye Biotechnology Co., Ltd. (Shanghai, China).

### 2.2. Cell Culture

The cells were cultivated in a culture incubator (37 °C, 5% CO_2_) in 89% RPMI 1640 media spiked with 10% fetal bovine serum and 1% dual antibody (100 U/mL penicillin and 100 μg/mL streptomycin). Cell passage was carried out according to the growth density, and then we proceeded to the next experiment after the cell state stabilized.

### 2.3. Assessment of the Morphology of LPS-Induced RAW264.7 Cells

A 6-well plate was inoculated with RAW264.7 cells (9 × 10^5^ cells/mL), which were then treated with DQTF and GYTR (100, 200, and 400 μg/mL) after 4 h of incubation at 37 °C. The control and model groups were treated with 2 mL RPMI 1640 medium. After incubation for 2 h, except for the control group, LPS (1 μg/mL) was applied to the other experimental group separately. After 10 h of intervention in the incubator, we observed the cell morphology of each group with an inverted microscope (Olympus CKX41, Shanghai Puh Optoelectronics Technology Co., Ltd., Shanghai, China) and photographed them.

### 2.4. Determination of TNF-α and IL-6 Levels in RAW264.7 Cells

With reference to Section 2.3, each group was dosed separately and incubated for 24 h. Then, they were centrifuged (2000–3000 rpm/min for 20 min), and we collected the cell supernatant. The cytokine levels were detected using ELISA assay kits in the supernatant [31].

### 2.5. Determination of Antioxidant Enzyme Levels in RAW264.7 Cells

After incubation for 24 h, the antioxidant enzyme levels in the cell culture supernatant were determined with kits according to their instructions.

### 2.6. Preparation of PeCTHC and PeTHC

An amount of muscle tissue from *Ruditapes philippinarum* was homogenized by mixing it with deionized water at a ratio of 1:3 (*w*/*v*). Pepsin (enzyme concentration, 2500 U/g; pH, 2.0; time, 2 h; temperature, 37 °C), trypsin (enzyme concentration, 3000 U/g; pH, 8.0; time, 4 h; temperature, 37 °C), and chymotrypsin (enzyme concentration, 5000 U/g; pH, 8.0; time, 4 h; temperature, 37 °C) were chosen for sequential hydrolysis under their own optimum. At the end of the enzymatic hydrolysis, the enzymes were inactivated (boiling water bath at 100 °C for 20 min), followed by centrifugation (10,000 rpm/min at 4 °C for 15 min). Subsequently, the supernatant was freeze-dried to obtain pepsin–chymotrypsin–trypsin composite enzymatic hydrolysis of clams (PeCTHC). The preparation of pepsin–trypsin composite enzymatic hydrolysis of clams (PeTHC) is explained above.

### 2.7. Determination of Short Peptide Yields of PeCTHC and PeTHC

We followed the method of Bai [32], with a slight modification. The products of enzymatic hydrolysis and TCA (10%, *w*/*v*) were combined at a ratio of 1:1 (*v*/*v*), left to stand for 10 min, and centrifuged to obtain the supernatant. Subsequently, the supernatant was mixed with bis-urea at a 2:3 (*v*/*v*) ratio. After standing for 10 min at 60 °C, the absorbance value was detected at 540 nm. To calculate the short peptide concentration using a standard curve prepared from glutathione standards. The concentration of protein in the enzyme hydrolysis products was measured according to a BCA assay kit, and we calculated the short peptide yield according to Formula (1):

(1) $$Y\left(\%\right)=\frac{C\times V}{W}\times 100\%$$

where *Y* is the short peptide yield, C is the concentration of short peptides in the enzymatic hydrolysis product, V is the total volume of TCA, and W is the concentration of protein in the enzymatic hydrolysis product.

### 2.8. Animals and Experimental Design

Thirty healthy adult C57BL/6J male mice were provided by ZhuHai Bestest Biotechnology Co., Ltd. The experiments were approved by the Animal Ethics Committee of Guangdong Ocean University, (approval number: GDOU-LAE-2022-021; approval date: 10 September 2022). Before the experiment, all animals were provided with water and fed freely for 7 days, and the room was kept at 23 ± 3 °C.

**Figure 1:**
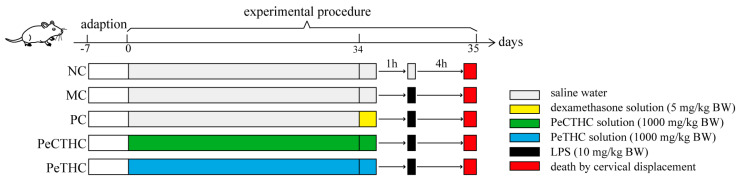
Experimental design in mice.

A total of thirty mice were randomly divided into five groups (*n* = 6) according to body mass. In the PeCTHC and PeTHC groups, the mice were orally administered with PeCTHC and PeTHC solutions (1000 mg/kg BW) once a day, respectively, while in the normal control group (NC), the model control group (MC), and the positive control group (PC), the mice were given an equal volume of saline water once a day for 5 weeks. On the last day of the experiment, the mice in all groups were orally administered as originally planned except for the PC group, which was injected with dexamethasone solution (5 mg/kg BW). After 1 h, saline was injected intraperitoneally in the NC group, and LPS (10 mg/kg BW) was injected intraperitoneally for 4 h in the remaining groups (Figure 1). Subsequently, the mice were weighed before being executed by cervical displacement, and blood samples were collected from the eyes. The livers and spleens were removed and weighed, and the organ indices were calculated, followed by storing the livers at −80 °C. The formula for calculating the organ indices is as follows (2):

(2) $$A\left(\%\right)=\frac{B}{M}\times 100\%$$

where *A* is the organ indices, B is the organ weight, and M is the mouse body mass.

### 2.9. Determination of Serum Pro-inflammatory Cytokines and NO Levels

We allowed the blood samples to naturally coagulate at room temperature for 30 min; then, they were centrifuged to collect the serum. Subsequently, we used a NO kit to measure the NO level in the serum, and the concentrations of pro-inflammatory cytokines were determined using commercial ELISA kits.

### 2.10. Complete Blood Cell Analysis

A blood cell analyzer (BV-5000Vet, Shenzhen Mindray Animal Medical Technology Co., Ltd., Shenzhen, China) was used to perform blood cell counts, including the total white blood cell (WBC), total red blood cell (RBC), ratio of lymphocytes (LYM), granulocyte count (GRAN), and platelet count (PLT).

### 2.11. Histopathologic Observation of Mouse Liver

To observe the state of the mouse liver tissue, HE staining was used. Briefly, we used 4% paraformaldehyde to fix the liver tissues, embedded them in paraffin, and stained them with HE, followed by observation with a light microscope (Eclipse E100, Shanghai Nikon Instruments Co., Ltd., Shanghai, China).

### 2.12. Determination of SOD, GSH-PX, and MDA Levels in Mouse Liver Tissue

The liver tissue was mixed with saline water at a ratio of 1:9 (*w*/*v*) and ground thoroughly to obtain the supernatant after centrifugation. The indicators of oxidative damage in the liver were determined using SOD, GSH-PX, and MDA kits.

### 2.13. Statistical Analysis

All data were shown as the mean ± standard deviation (SD). Statistical analysis was performed using SPSS 26.0 software (IBM, USA), and one-way analysis of variance (ANOVA) was used to analyze the experimental data. The least-significant difference (LSD) method was used to compare the differences among the groups. *p* < 0.05 was considered statistically significant, *p* < 0.01 was considered highly statistically significant, and *p* < 0.001 was considered extremely highly statistically significant.

## 3. Results and Discussion

### 3.1. Effects of DQTF and GYTR on Cell Morphology

**Figure 2:**
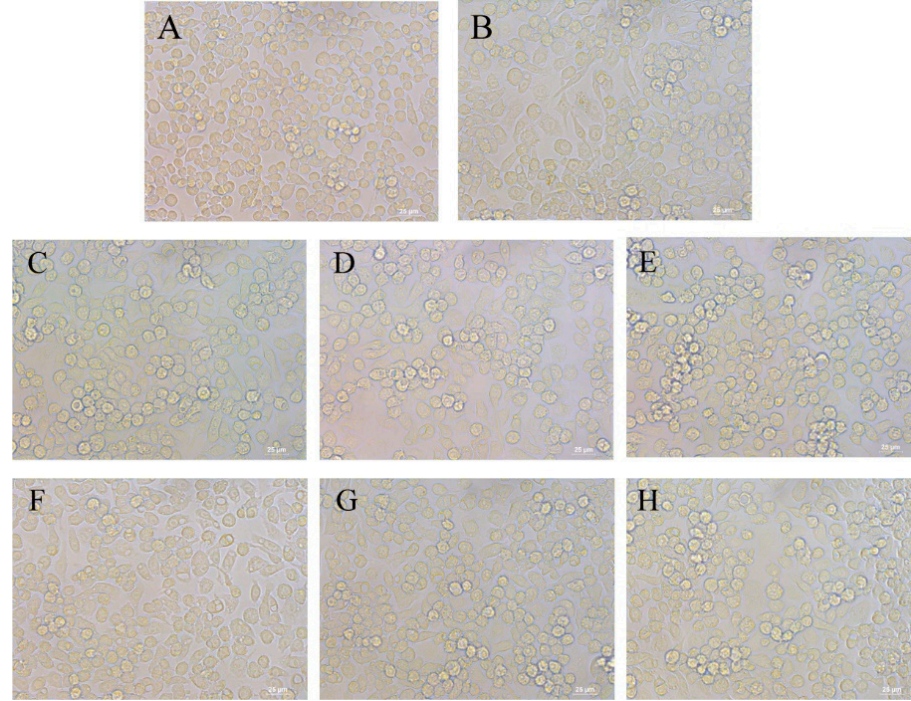
Effects of DQTF and GYTR on morphological changes in RAW264.7 cells. (**A**) Control; (**B**) Model; (**C**–**E**) 100, 200, and 400 μg/mL of DQTF; and (**F**–**H**) 100, 200, and 400 μg/mL of GYTR.

The morphology of RAW264.7 cells is altered by cellular activity and function. When cells are stimulated by LPS or other signaling factors, they undergo differentiation and grow pseudopods [33]. As shown in Figure 2, normal RAW264.7 cells were round and full in shape, as observed with inverted fluorescence microscopy (Figure 2A). In contrast, the morphology of the cells was induced to be pike-like angular (cell body hypertrophy and irregularity), with a reduced number of cells and the production of pseudopods and cytoplasmic granules (Figure 2B). This could be caused by LPS stimulation, as analogical studies have reported previously [34]. We used 100, 200, and 400 μg/mL of DQTF and GYTR to treat the LPS-induced cells. The findings demonstrate that the extent of cell differentiation was improved after the administration of different concentrations of DQTF, GYTR, and co-culture with LPS. Additionally, better cell morphology improvement was shown at high concentrations, making its morphology more convergent to that of the blank control group (Figure 2). According to a report, mung bean protein hydrolysate can improve LPS-induced cell differentiation and morphology [4], which is similar to the results of our study. According to the above analysis, DQTF and GYTR can improve inflammatory cell morphology.

### 3.2. Effects of DQTF and GYTR on TNF-α and IL-6 Levels in RAW264.7 Cells

TNF-α and IL-6 are important pro-inflammatory cytokines that play important roles in mediating anti-tumor responses and regulating immune function, and anti-inflammation [31]. In particular, TNF-α is a pro-inflammatory cytokine secreted by activated macrophages that activates the nuclear factor (NF)-κB signaling pathway, which regulates an array of inflammatory factor secretions [35]. In chronic inflammation and autoimmunity, activated macrophage produces IL-6 continues to play an important role [36]. Therefore, pro-inflammatory cytokine levels can be modulated to attenuate the inflammatory response.

**Figure 3:**
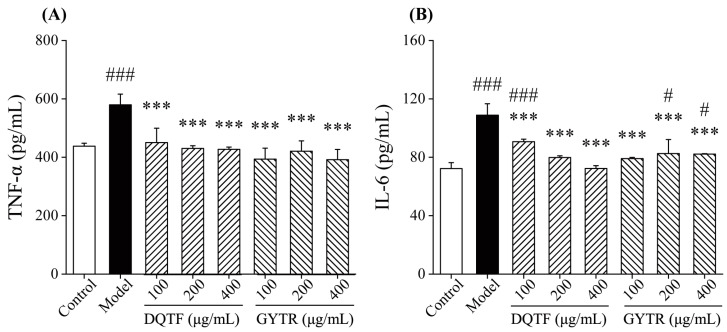
Effects of DQTF and GYTR on TNF-α (**A**) and IL-6 (**B**) production in RAW264.7 cells. Data are expressed as mean ± SD (*n* = 3). # *p* < 0.05 and ### *p* < 0.001, vs. control group; *** *p* < 0.001, vs. model group.

The concentrations of TNF-α and IL-6 in RAW264.7 cells were measured, and the outcomes are shown in Figure 3. The TNF-α and IL-6 concentrations of the model group were highly markedly increased (*p* < 0.001, vs. control group). This is due to the fact that LPS stimulation causes a large amount of pro-inflammatory cytokines to be produced in the cells [15]. In this study, 100, 200, and 400 μg/mL of DQTF and GYTR could reduce the impact of LPS. As depicted in Figure 3A, the TNF-α level in the DQTF and GYTR groups was distinctly lower than that in the model group, which was close to that of the control group. Similarly, the production of IL-6 decreased significantly (*p* < 0.001, vs. model group, Figure 3B). It has been reported that abalone peptide could effectively inhibit LPS-induced transcript levels of pro-inflammatory cytokines in RAW264.7 cells [15]. Our results suggest that DQTF and GYTR had a similar effect on cytokine production.

### 3.3. Effects of DQTF and GYTR on LPS-Induced Oxidative Damage in RAW264.7 Cells

**Figure 4:**
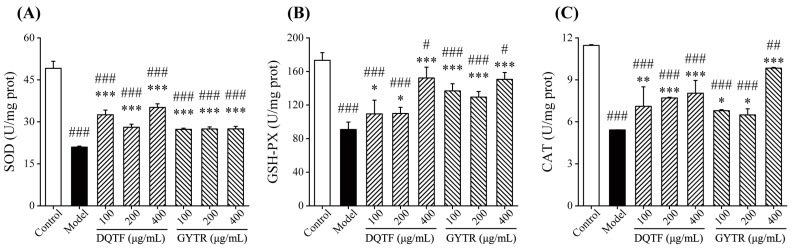
Effects of DQTF and GYTR on antioxidant enzyme levels in RAW264.7 cells. (**A**) SOD, (**B**) GSH-PX, and (**C**) CAT. Data are expressed as mean ± SD (*n* = 3). # *p* < 0.05, ## *p* < 0.01, and ### *p* < 0.001, vs. control group; * *p* < 0.05, ** *p* < 0.01, and *** *p* < 0.001, vs. model group.

The main intracellular enzyme systems include CAT, SOD, and GSH-PX, which are known as the protective enzyme systems of cells that defend against inflammatory responses by maintaining intracellular oxidative homeostasis [37]. As depicted in Figure 4, SOD, GSH-PX, and CAT enzymatic activities in the control groups were detected to be 49.15, 173.35, and 11.46 U/mg, while the levels of the model groups were reduced to 20.98, 90.86, and 5.42 U/mg, respectively. After administration with 100, 200, and 400 μg/mL of DQTF or GYTR, all the antioxidant enzyme levels in the cells were markedly elevated in a dose-dependent fashion (*p* < 0.001), and the maximum effect was observed in the group with 400 μg/mL of peptides. Similar results were reported where intracellular oxidative homeostasis was disrupted, and the activity of its protective enzymes was decreased after induction with LPS [16]. Similarly, several studies have reported that anti-inflammatory peptides enhance intracellular antioxidant mechanisms by increasing antioxidant enzyme levels [38,39]. The above results indicate that DQTF and GYTR ameliorated LPS-induced cellular oxidative damage and, thus, alleviated inflammation.

### 3.4. Short Peptide Contents of PeCTHC and PeTHC

**Figure 5:**
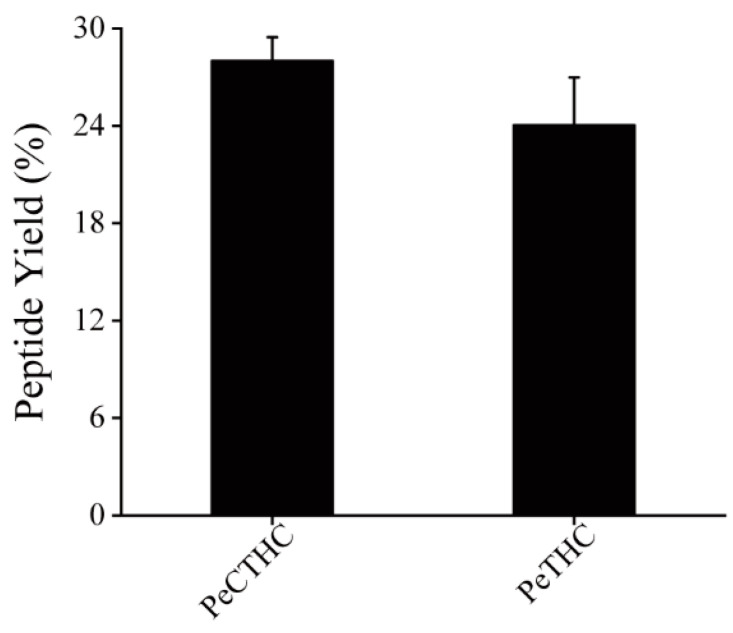
Short peptide yields of PeCTHC and PeTHC. Data are expressed as mean ± SD (*n* = 3).

To investigate the anti-inflammatory effect of *Ruditapes philippinarum* peptides (RPPs) in lipopolysaccharide-induced mice, we selected two enzymatic hydrolysis methods to prepare two enzymatic hydrolysates (PeCTHC and PeTHC) from the muscle tissue following the same method as virtual enzymolysis. Short peptides are peptides with molecular weights between 180 and 1000, which have the advantages of small molecular weight and simple structure. Furthermore, short peptides are easy to digest and absorb. Previous studies have confirmed that proteins can effectively release peptides after enzymatic hydrolysis via biological proteases [40]. However, the peptides released by different enzymatic methods differ in content and quantity due to the specificity of proteases, followed by the yield of short peptides, which is an important indicator reflecting the effect of enzymatic hydrolysis and is closely related to the extent of hydrolysis [41]. It can be seen in Figure 5 that PeCTHC had a higher short peptide yield of 28.01 ± 1.45%. This is probably due to the fact that the three enzymes work together at more cleavage sites, resulting in the release of more short peptides. These results also indicate that there were still more small molecular peptides when the *Ruditapes philippinarum* proteins were under the action of gastrointestinal digestive enzymes.

### 3.5. Effects of PeCTHC and PeTHC on Organ Indices in Mice

**Figure 6:**
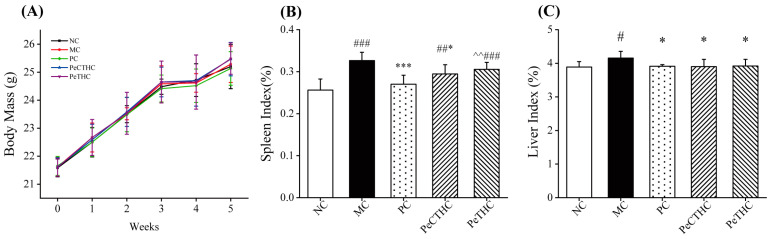
Effects of PeCTHC and PeTHC on body weight and organ indices in mice. (**A**) Body mass, (**B**) spleen index, and (**C**) liver index. Data are expressed as mean ± SD (*n* = 6). # *p* < 0.05, ## *p* < 0.01, and ### *p* < 0.001, vs. NC group; * *p* < 0.05, and *** *p* < 0.001, vs. MC group; ^^ *p* < 0.01, vs. PC group.

During the test period, the mice maintained a consistent increase in body weight, which was recorded once a week, and no significant difference in body weight was observed among the groups of mice (Figure 6A).

The organ indices are able to reflect the development of the organ and the degree of damage [42]. The liver is one of the most common organs that responds to acute inflammation, while the spleen is a major immune organ [43,44]. Thus, the liver and spleen indices of the mice were measured, and the outcomes are displayed in Figure 6B,C. Compared to the NC group, the liver and spleen indices were markedly enhanced in the MC group, indicating successful modeling of acute inflammation. With the administration of enzymatic hydrolysates, the liver index in the PeCTHC and PeTHC groups decreased (*p* < 0.05, vs. MC group), which was close to that of the PC group (Figure 6C). On the other hand, the spleen index was also distinctly lower in the PeCTHC group (*p* < 0.05, vs. MC group, Figure 6B). Nevertheless, the PeTHC group was not significantly different. In conclusion, PeCTHC and PeTHC were able to reduce the liver and spleen indices, and it was initially speculated that their pathway to regulate LPS inflammatory injury in mice may be related to immunomodulation.

### 3.6. Effects of PeCTHC and PeTHC on NO and Cytokines Levels in Serum of Mice

As a key inflammatory target, NO is a well-recognized indicator of inflammation in animal models and regulates inflammatory activity through inducible nitric oxide synthase (iNOS) [45,46,47].

**Figure 7:**
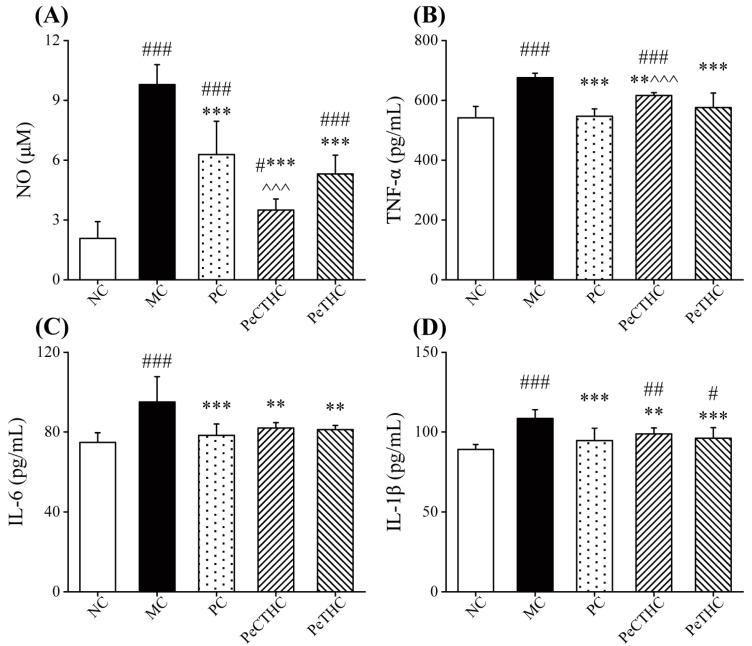
Effects of PeCTHC and PeTHC on serum inflammatory factors in mice. (**A**) NO levels, (**B**) TNF-α, (**C**) IL-6, and (**D**) IL-1β. Data are expressed as mean ± SD (*n* = 6). # *p* < 0.05, ## *p* < 0.01, and ### *p* < 0.001, vs. NC group; ** *p* < 0.01, and *** *p* < 0.001, vs. MC group; ^^^ *p* < 0.001, vs. PC group.

As depicted in Figure 7A, the NO concentration was significantly elevated in the MC group (*p* < 0.001, vs. NC group) when treated with LPS, while the serum NO concentration decreased distinctly with the administration of PeCTHC and PeTHC (*p* < 0.001, vs. MC group). Similarly, sturgeon muscle protein peptides reduced NO production to alleviate LPS-induced inflammation [48]. It can be concluded that PeCTHC and PeTHC can attenuate inflammation by deterring NO production.

Cytokines are a class of endogenous peptides, produced by cells of the immune system, with many powerful biological effects, and they mediate immune and inflammatory responses [31]. When LPS stimulates an organism, the innate immune response in vivo is activated, eliciting an inflammatory response that results in a significantly increased expression of pro-inflammatory cytokines [14,49]. In order to evaluate the influence of PeCTHC and PeTHC on pro-inflammatory cytokines, commercial ELISA kits were used. The findings show that the TNF-α, IL-6, and IL-1β levels in the MC group were significantly enhanced (*p* < 0.001, vs. NC group, Figure 7B–D). Notably, LPS stimulation causes the liberation of pro-inflammatory cytokines, which, in turn, trigger inflammation in mice.

Furthermore, diet intervention with PeCTHC and PeTHC before acute inflammation (LPS stimulation) could effectively reduce the concentrations of TNF-α, IL-6, and IL-1β (*p* < 0.01 or *p* < 0.001, vs. MC group). Moreover, no significant differences in IL-6 and IL-1β compared to the PC group were found. Similar to our results, previous research demonstrated that oral administration of corn silk peptide with anti-inflammatory effects reduced cytokine levels in mice with LPS-induced inflammatory injury mice [50]. Our results further demonstrate that the advanced intake of PeCTHC and PeTHC can attenuate LPS-induced acute inflammatory injury in mice by decreasing the contents of pro-inflammatory cytokines in the serum.

### 3.7. Effects of PeCTHC and PeTHC on Blood Indices in Mice

**Figure 8:**
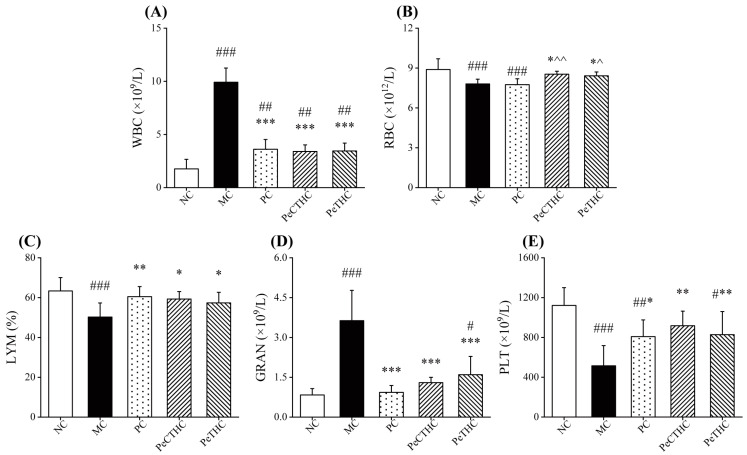
Effects of PeCTHC and PeTHC on blood indices in mice. (**A**) WBC, (**B**) RBC, (**C**) LYM, (**D**) GRAN, and (**E**) PLT. Data are expressed as mean ± SD (*n* = 6). # *p* < 0.05, ## *p* < 0.01, and ### *p* < 0.001, vs. NC group; * *p* < 0.05, ** *p* < 0.01, and *** *p* < 0.001, vs. MC group; ^ *p* < 0.05 and ^^ *p* < 0.01, vs. PC group.

Inflammation was evaluated through the measurement of WBC, RBC, LYM, GRAN, and PLT levels in the blood. WBC and GRAN were significantly elevated, while RBC, LYM, and PLT were significantly reduced after the intraperitoneal injection of LPS in mice (vs. NC group, Figure 8). This shows that LPS stimulation altered the blood indices in the mice. Indeed, when LPS stimulates the organism, the leukocyte system is activated, and the number of WBCs increases [51]. Similarly, a significant GRAN is produced, leading to an exacerbated inflammatory response [52]. Moreover, RBC, PLT, and LYM decrease after the organism is stimulated by LPS, which results in inflammation [53,54].

However, when PeCTHC and PeTHC were given, the amount of WBCs and the GRANs significantly decreased (*p* < 0.001, vs. MC group, Figure 8A,D). Similarly, RBC, LYM, and PLT tended to return to normal levels (vs. MC group, Figure 8B,C,E), which is consistent with the results in the PC group. This implies that early consumption of PeCTHC and PeTHC can attenuate LPS-induced acute inflammatory damage by modulating blood indices. Previous studies have found that the Scutellaria baicalensis–forsythiae pairing also improved blood indices in mice with LPS-induced acute lung injury, which resulted in the alleviation of pneumonia [5]. The above results suggest that substances with anti-inflammatory activity can ameliorate inflammatory injury by regulating blood indices.

### 3.8. Effects of PeCTHC and PeTHC on Histopathology of Mouse Liver

LPS stimulation leads to tissue damage and pathological changes in the livers of mice [55]. As shown in Section 3.5, the liver index of mice stimulated by LPS was significantly elevated. In order to visualize the histopathological changes in the mouse liver, HE dyeing was performed on the liver tissue.

**Figure 9:**
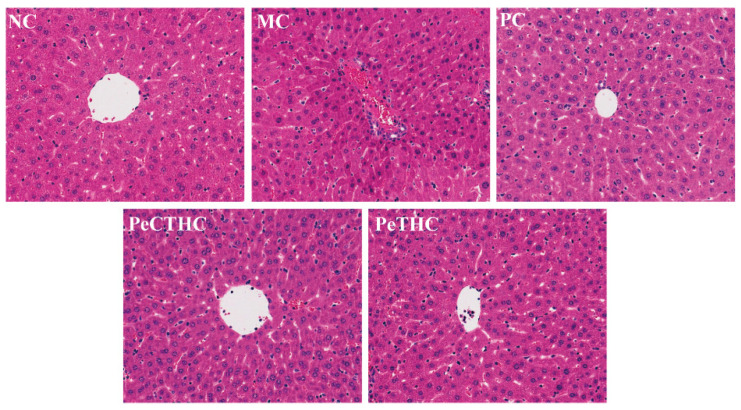
Effects of PeCTHC and PeTHC on histopathology of mouse liver (200×).

In Figure 9, the histologic morphology of the liver is illustrated. The liver tissues of the mice in the NC group were intact, exhibiting uniform distribution of cells and hepatic cords, without tissue necrosis or inflammatory lesions, based on the histopathological findings. Conversely, the MC mice exhibited swollen livers with cellular necrosis and irregularly distributed hepatic cords with inflammatory symptoms. In addition, the PeCTHC and PeTHC groups showed a reduced number of hepatic inflammatory cells with neater hepatic cords and alleviation of liver lesions (vs. MC group), which is similar to the results of the PC group. The same efficacy was also found in mice with inflammation that were administered AWRK6 (synthetic peptide) [56]. In summary, it can be seen that PeCTHC and PeTHC exhibit pre-protective efficacy on LPS-induced liver injury.

### 3.9. Effects of PeCTHC and PeTHC on SOD, GSH-PX, and MDA Levels in Mouse Liver

**Figure 10:**
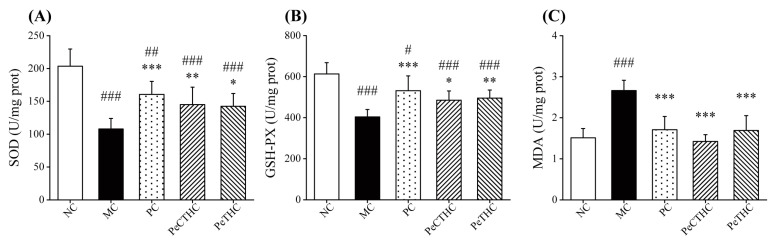
Effects of PeCTHC and PeTHC on (**A**) SOD, (**B**) GSH-PX, and (**C**) MDA levels in mouse liver. Data are expressed as mean ± SD (*n* = 6). # *p* < 0.05, ## *p* < 0.01, and ### *p* < 0.001, vs. NC group; * *p* < 0.05, ** *p* < 0.01, and *** *p* < 0.001, vs. MC group.

Oxidative damage is considered to be the main cause of most inflammatory diseases [12]. Previously, SOD and GSH-PX were found to be important antioxidant substances in organisms. SOD was able to convert the superoxide anion into hydrogen peroxide, thus scavenging free radicals and maintaining the oxidative balance of the organism. Furthermore, GSH-PX prevents oxidative damage to cell membranes by catalyzing the breakdown of hydrogen peroxide into water. Both of them work together to maintain the oxidative balance in the organism [57,58,59]. To explore the effects of PeCTHC and PeTHC on the levels of antioxidant enzymes, both SOD and GSH-PX were measured in mouse livers. In the MC group, SOD and GSH-PX activities in the liver were significantly decreased (*p* < 0.001, vs. NC group, Figure 10A,B). Instead, similar to the PC group, the early feeding of PeCTHC and PeTHC inhibited the effects of LPS and reduced the decline in SOD and GSH-PX concentrations.

As for MDA, it is a major product of lipid oxidation reactions, which may reflect the extent of lipid peroxidation in vivo [60]. As displayed in Figure 10C, the MDA level was markedly higher after LPS intervention. Nevertheless, the MDA content was reduced after either PeCTHC or PeTHC treatments (vs. MC group). As expected, PeCTHC and PeTHC enhanced antioxidant enzyme activities and reduced lipid oxidation, resulting in lower MDA production. Furthermore, Mao et al., found that LPS reduced the antioxidant capacity of mouse livers, leading to oxidative stress and liver injury. In contrast, protein components from antler-based powder significantly restored the antioxidant levels in mouse liver [61], which coincides with our research.

In conclusion, PeCTHC and PeTHC showed protective effects against LPS-induced oxidative damage in mouse livers, which could attenuate oxidative injury in them by restoring antioxidant enzyme activities and decreasing the production of malondialdehyde, thereby reducing LPS-induced inflammatory injury in mice.

## 4. Conclusions

In the current study, we validated the anti-inflammatory activities of DQTF and GYTR by constructing a model of LPS-induced inflammatory injury in RAW264.7 cells. It was found that DQTF and GYTR (100–400 μg/mL) significantly improved cell morphology and decreased TNF-α and IL-6 levels in LPS-injured cells. In addition, they distinctly increased cellular antioxidant enzyme levels, improved cellular oxidative damage, and played a protective role in the LPS-induced cellular inflammation model. Furthermore, PeCTHC and PeTHC were obtained via the enzymatic hydrolysis of *Ruditapes philippinarum* and then subjected to in vivo animal experiments. The findings showed that PeCTHC and PeTHC decreased the levels of pro-inflammatory cytokines and NO in the serum of mice while also improving both the blood indices and the morphology of the organs of mice. Moreover, they also attenuated LPS-induced oxidative damage in the liver, thus protecting against LPS-induced acute inflammation in mice. In conclusion, both in vivo and in vitro experiments confirmed the anti-inflammatory activity of RPPs and the protective effect of RPPs against LPS-induced acute inflammation. These findings may serve as a theoretical basis for investigations into RPPs in the field of functional foods.

## Data Availability

The original contributions presented in the study are included in the article, further inquiries can be directed to the corresponding author.
